# Promoting Healthcare Sustainability in Developing Countries: Analysis of Knowledge Management Drivers in Public and Private Hospitals of Pakistan

**DOI:** 10.3390/ijerph16030508

**Published:** 2019-02-12

**Authors:** Jawad Karamat, Tong Shurong, Naveed Ahmad, Sana Afridi, Shahbaz Khan, Kashif Mahmood

**Affiliations:** 1 School of Management, Northwestern Polytechnical University, Xi’an 710072, Shannxi, China; jawad@mail.nwpu.edu.cn (J.K.); stong@nwpu.edu.cn (T.S.); 2 Department of Pathology, School of Basic Medical Science, Xi’an Jiaotong University, Xianning West Road, Xi’an 710049, Shaanxi, China; sanaafridi@stu.xjtu.edu.cn; 3 School of Automation, Northwestern Polytechnical University, Xi’an 710072, Shannxi, China; shahbaz@mail.nwpu.edu.cn; 4 Department of Management Science, Bahria University, Islamabad 44220, Pakistan; kashifmahmood81@gmail.com

**Keywords:** healthcare sustainability, drivers, knowledge management, interpretive structural modeling, developing countries, Pakistan

## Abstract

Investing in a sustainable future has no alternative; the healthcare sector in developing countries has failed to achieve sustainability objectives. Knowledge management (KM) is a concrete application of sustainability in healthcare, as organizations (hospitals) that manage their knowledge assets will gain sustainable competitive advantage. Several organizations in developed countries are moving towards the adoption of knowledge management so that they can manage their knowledge well and improve their performance. Due to the effective implementation of KM in developed countries, developing countries are also considering adopting KM in their healthcare. In this study, an attempt has been made to identify the drivers of KM adoption in public and private hospitals of Pakistan. With the help of an extensive literature review and expert opinion, the drivers were identified and a hierarchical structure was developed. Nineteen drivers were identified and screened out by experts. The experts identified the contextual relationships between the drivers during a brainstorming session. The hierarchical model of the drivers for KM in the healthcare of Pakistan was eventually developed using interpretive structural modeling (ISM). The structure has 10 levels, in which “developed competitive advantage” formed the foundation of the structure and “job creation” and “improvement in the reputation of healthcare” formed the topmost level. The “Matrices d’Impacts Croises Multiplication Appliqué a un Classement” (MICMAC) analysis classified the drivers by categorizing them according to their driving and dependence powers. One driver is identified as autonomous, six drivers as dependent, seven drivers as linkage, and five drivers as independent. The analysis of KM drivers will provide a good understanding of the interdependence and interactions between them and support the effect adoption of KM in developing countries especially in Pakistan.

## 1. Introduction

Healthcare sustainability has captured great attention in the recent era globally, in view of economic and social crises that developing countries are recently facing. Therefore, it is necessary to generate value and integrate sustainability in the healthcare sector of developing countries—always considering the importance of all stakeholder demands [1]. Sustainable healthcare systems can be defined as “Systems which deliver high quality care and improved public health without exhausting natural resources or causing severe ecological damage” [2]. Further, Fineberg [2] attributed the triple-A concept with sustainable healthcare system, which includes affordability, acceptability, and adaptability for all stakeholders including patients, employees, employers, healthcare agencies, and government. Coulson-Thomas [3] indicated knowledge management (KM) as a quicker, improved, and affordable route for greater organizational performance, especially in service-based organizations. Knowledge has become an important resource and asset to become strategically competitive in current era [4]. Therefore, KM does not only provide an effective solution to manage knowledge in a knowledge intensive service industry, but also contributes towards sustainable development by effectively manage organizational resources [5]. Realizing that healthcare needs are not static and it needs to improve itself in a competitive environment is important. KM has been contributing extensively in managing organizational resources, improving organizational performance, and gaining sustainable competitive advantage in different areas. KM, as one of the sustainable organizational practices and intellectual capital [6], can also support healthcare to gain sustainable competitive advantage [7]. Due to globalization, there is intense competition between the service-based industries and high reliance on knowledge; knowledge needs to be digitized, reliable, inexpensive, easily available, and speedy, it is considered a key intangible resource [8]. Knowledge management (KM) aids people in collaborating, innovating, and making correct decisions efficiently [9]. It helps healthcare professionals in making high-quality decisions based on knowledge retained through KM; it develops positive attitude among the hospitals staff and the way they work [10]. The staff consists of people from different backgrounds, that get together and work, using their abilities to achieve a specific goal that is to create, share, gather, and to leverage knowledge [11]. The staff is encouraged to share ideas, information, and knowledge which changes their way of thinking, and they focus on learning. Organizations gain a sustainable competitive advantage by using KM for managing, creating, capturing, sharing, and leveraging knowledge. The advancements in Information Technology (IT), databases, and semantic software have enabled the development and effective application of KM [12]. On the other side, the KM creates also an important role in achieving sustainability in public based organizations, especially in healthcare, as KM does not only provide support to hospitals in decision making, but also ensures long-term quality care for patients. In case, KM is not implemented and sustained appropriately, it may lead to poor quality of care and quality of life [13]. Therefore, evaluating KM implementation as a sustainable solution to healthcare is necessary. 

Most of the healthcare sector organizations of developed countries, America, Canada, Europe, etc. have already shifted towards KM to increase collaboration and sharing of information to make healthcare organizations sustainable and able to provide optimal healthcare performance [10,12]. The performance is improved by coordination and cooperation of several partners in recording and transmission of daily healthcare activities knowledge. There is a great reliance on knowledge and evidence-based medicine (EBM) in the healthcare sector [14]. EBM helps the public healthcare sector organizations in optimized decision-making based on evidence from well-defined and well-conducted data generated by knowledge, and it considerably improves performance. Goldsmith [15] mentioned that decision making in healthcare is very critical, it affects the patient care greatly. Other than performance, KM also increases the accountability of the healthcare organizations; the availability of information at the appropriate time reduces the chance of medical errors. These reduced medical errors result in fewer lawsuits [16]. After observing the positive changes brought about by KM in developed countries, some developing countries are also considering adopting KM in the healthcare sector.

Among the developing countries, Iran has worked the most on adoption of KM in its healthcare sector [17,18,19]. Pakistan, being influenced by Iran, has also shown great interest in KM implementation in its public and private hospitals. Currently, KM implementation is in its infancy. The healthcare of Pakistan has suffered greatly due to increase in diseases [20], population growth [21], limited resources [22], and performance issues [23], and is now looking towards KM to resolve these issues and to improve performance. Therefore, the objective of this study is to identify the drivers of KM in the healthcare industry of developing countries in the context of Pakistan. This study was considered for four main reasons; firstly, there is scarcity on literature related to KM implementation in the healthcare sector, specifically in developing country e.g., Pakistan. Secondly, most of the earlier studies focused on the KM implementation in private and profit oriented businesses, however, the non-profit and public oriented organizations such as hospitals (healthcare) lacks the literature on KM implementation [24], which is one of the major contributions towards the KM literature. Thirdly, despite the multiple benefits attached to KM, drivers to promote KM are not extensively discussed in the healthcare. Fourth, drivers to KM implementation need to be discussed through scientific research and with an established methodology. This study employs the Fuzzy Delphi Method (FDM) to shortlist the drivers collected from extensive literature review and then applies the Interpretive Structural Modeling (ISM) technique for developing the relationship between different drivers and represents them in a hierarchical form [25]. 

Many researchers of various different fields have used the integrated methodology of ISM and “Matrices d’Impacts Croises Multiplication Appliqué a un Classement” (MICMAC) in their analysis such as engineering [26,27], supply chain management [28,29], total quality management [30,31], reverse logistics [32,33], and knowledge management [34,35]. However, the application of ISM in healthcare is very limited [36,37]. This paper is organized into five sections, starting with the introduction in Section 1. The theoretical perspective is given in Section 2. The research methodology is explained in Section 3. Section 4 presents the research analysis and results, and it is concluded in Section 5.

## 2. Theoretical Perspective

### 2.1. Knowledge Management and Healthcare

Dependence on knowledge leads the healthcare sector to implement an effective system to manage knowledge properly as new knowledge is continuously being generated [8]. The developed countries realized the importance of knowledge early on and moved towards the adoption of knowledge management. Countries such as USA, Canada, UK, and the European Union are using KM [10] in their healthcare. Developing countries are now also looking towards the adoption of knowledge management in their healthcare. Currently, Iran is among the developing countries that has made the most effort [17,18,19]. 

Currently, there are several definitions of knowledge management within the healthcare; this makes it rather confusing to understand. Some experts, Despres and Chauvel [38] consider KM to be a very important issue and at the same time find it intellectually elusive. Traditionally, KM can be defined as an intellectual capital and considered as an important organizational intangible asset. It is the accumulation of all information resources driving an organizational towards improved profitability, gaining new markets, improved new products or services, processes, or otherwise grow the business exponentially. However, they also describe that it is very difficult to define knowledge in clear terms without it being abstract; this makes knowledge seem like “everything and nothing”. Later, Nonaka [39] introduced the concept of KM while working on organizational knowledge creation processes and defined it as the process of coordination, transformation, and transfer of knowledge. KM aims to identify the corporate knowledge and to improve coordination by facilitating the communication of knowledge to ensure it reaches the people that created it, as well as the people that require it [40]. KM also helps in the process of creating, storing, transferring, and application of knowledge within organizations [41]. 

Many researchers and experts have accepted the importance of knowledge, and it is said to be the source of sustainable competitive advantage [42,43]. Nonaka [44] and Sveiby [45] expressed that there are two types of knowledge: tacit and explicit. Tacit is un-codified knowledge that is learned via experience, while explicit is codified and learned by books, manuals, researches, etc. The role of KM is to transform tacit into explicit knowledge and vice versa [44]. 

Knowledge management has become an important tool for health care and medical practice because the ability of the human brain to recall and process a large amount of knowledge is limited. Most of the knowledge that the healthcare professional retains during his service becomes obsolete over time, and they need to learn new techniques to treat patients. Despite the knowledge of a doctor, the mind cannot process a large amount of information with numerable variables to develop proper diagnostic or treatment options based on individual patients’ characteristics. Doctors in modern times need a connection with a medical knowledge archive to keep updated and to apply knowledge properly to improve healthcare delivery [46]. 

### 2.2. Knowledge Management Implementation in Pakisani Healthcare

Pakistan ranks sixth in world according to population, with 212,742,631 people, it is a developing country in South Asia [47], and has a growth rate of 2%. The gross domestic product (GDP) of Pakistan is about $304.4 billion, growing at a rate of 5% per annum [48]. Pakistan is currently ranked 120 out of 190 countries in terms of healthcare performance [49]. Pakistan is facing many problems in its healthcare sector due to increase in diseases [20], population growth [21], limited resources [22], and performance issues [23]. Pakistan is suffering from both communicable and non-communicable diseases. The communicable diseases consist of HIV (0.36%), hepatitis B and C (5%), tuberculosis (2.29%), malaria (0.43%), diarrhea (4.65%), dengue (0.061%), etc. The non-communicable diseases consist of heart diseases (8%), diabetics (2.14%), eye diseases (1%), cancer (7.4%), trauma patients (3.64%), etc. (figures according to [50]). Pakistan has a high child mortality rate, about 66 to 46 out of 1000 die at birth, and 81 out of 1000 die before reaching the age of 5. The death rate of mothers during the birthing process is 178 per 100,000, out of which 51% were handled by skilled workers [51].

Realizing these problems in the healthcare sector, the Government of Pakistan (GoP) is considering the adoption of KM to improve performance, which it is currently at infancy stage in developing countries. KM can help in improving the performance by reducing the time spent on communication, recording, combining information of the patient because the information provided is often obsolete or unrecorded; it takes up 33% of doctors’ working hours, increases the cost [52], and also results in improper medical care for the patient. KM tools assist in the storing of knowledge and its retrieval to improve the decision-making process. It gives healthcare professionals the ability to access knowledge quickly while treating patients. KM also helps in introducing new information that helps in dealing with patients with uncommon problems [53], e.g., the first case of dengue in Pakistan was reported in 1994 in the largest city, Karachi, but when dengue reached the other regions of the country, the doctors of that area had no idea of the disease and how to cure it, resulting in the death of hundreds of people [54]. It is compulsory for the healthcare professionals of Pakistan to keep updating their knowledge to effectively learn modern techniques to deal with new and old diseases. Medical knowledge is being generated at a rapid pace and is estimated to be fourfold in a professional’s lifetime [55]. 

Due to the realization of multiple advantages of KM implementation, GoP is also interested in implementing it. KM will provide the healthcare sector with a sustainable competitive advantage [1,35]. Pakistan has developed the National Health Vision (2016) [56], in which it has set targets for 2025 in order to improve its healthcare. The targets have been approved by both the federal and provincial governments; Pakistan is trying its best to redeem its self. The healthcare sector organizations are now competing globally, and there is an increase in medical tourism as well [37]. By adopting KM in the healthcare of Pakistan, it can improve its service locally and can gain a share of the international competition as well.

### 2.3. Drivers of KM in the Healthcare

Many earlier studies have identified the drivers to KM adoption in different contexts. Du Plessis [57] identified several drivers of KM, gaining competitive advantage, managing knowledge as an asset, collaboration with other organizations, ease of transferring knowledge to other geographical locations, increased richness and reach of knowledge, reduction in knowledge attrition, increasing knowledge storage, and removal of internal inefficiencies. Du Plessis [58] stated that KM helps in adjusting to changes in the global market, aligns the goals and the objectives of the business, helps in managing the organizational behavior, creates a learning structure in the organization, improves collaboration over geographical boundaries, creates a social network within the organization, forms an incubator for innovation, prevents loss of knowledge, and improves knowledge and learning ethics. Lee and Choi [59] addressed collaboration, creating trust, learning within the organization, and decentralization for quick decision making as the main divers of KM. Du Plessis [60] revealed that creating a competitive advantage, collaboration with other organizations, involvement in innovation, improvement in the quality of knowledge, and the ability to share knowledge as the drivers to KM adoption. Yu et al. [61] mentioned three main drivers, organizational characteristics, information technology, and managerial support, as the main drivers for the effective implementation of KM. The organizational characteristic refers to organizational learning, improved communication, increased flexibility, and knowledge sharing intention. Information technology means the quality of the Knowledge management system and its functionality. The managerial support includes the reward system and team activity. Davenport et al. [62] stated effective decision making, improved administrative healthcare performance, reduced administrative cost, increased patient service level, reduction in patient expenses, increased organizational learning, the incubators of innovation, job creation opportunity, and decentralization in KM as the key drivers. Darko et al. [63] mentioned reduced administrative cost, improved service, better work environment, customer well-being, better performance, setting a standard, and adopting the best practices as the main drivers. Yu [64] identified effective decision making, reduced administrative cost, and reduction in patient expenses as important drivers of KM.

The drivers that were identified by a comprehensive literature review on KM in the healthcare are given in Table 1.

## 3. Research Methodology

This research uses two techniques: the Fuzzy Delphi Method (FDM) and the Interpretive Structure Modeling (ISM). The research methodology has been divided into three stages. First of all, a detailed literature review was performed to identify the key drivers. These drivers were shortlisted with the help of the FDM, and later analyzed using ISM and MICMAC analysis. 

**Figure 1:**
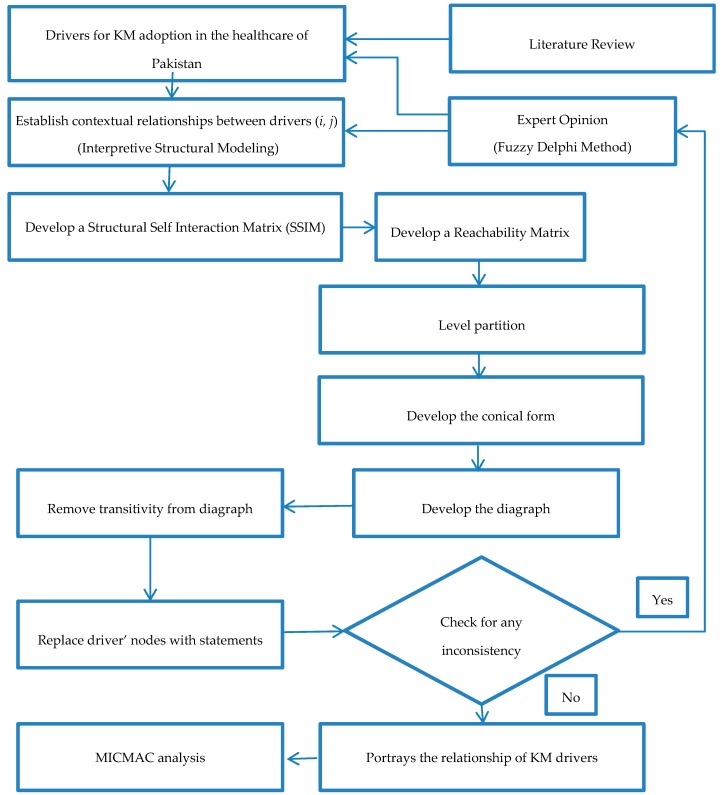
Research Methodology. KM: knowledge management; MICMAC: Matrices d’Impacts Croises Multiplication Appliqué a un Classement.

Stage 1: To identify the drivers of knowledge management in healthcare, a detailed literature review was performed, and many research articles were studied. These publications were searched using various databases such as Taylor & Francis, Web of Science, JSTOR, Emerald, PubMed, and Google Scholar. The keywords utilized to identify the relevant articles were, “Drivers,” “developing countries,” “Knowledge Management,” “benefits,” and “health care.” This resulted in a total of 80 articles from 30 journals. After removal of duplication, and irrelevant studies by studying the abstract, only 30 articles from 20 journals remained, along with two conferences and one book. Some of the famous journals considered for this study are Journal of Knowledge Management (4 Papers), European Management Journal (4 Papers), Journal of Management Information System (3 Papers) International Journal of Information Management (2 Papers), Information and Software technology (2 papers), Journal of Cleaner Production (2 Papers), and Information System management (1 Paper). The research methodology of this study is given in Figure 1.

### 3.1. Fuzzy Delphi Method (FDM)

Stage 2: the Delphi Method (DM) is most widely used in social sciences, engineering, management, and health sciences research. DM utilizes expert opinion and is easy to practice due to lessor criteria for expert selection. DM has some disadvantages, e.g., high execution cost, the possibility of low convergence, and filtering particular expert opinions [78]. Therefore Murry et al. [79] integrated DM with the fuzzy theory to remove the ambiguity and vagueness. Ishikawa et al. [80] improved the DM further by including the fuzzy theory and developing the max-min Fuzzy Integration algorithms to determine the prevalence of computers in the future. This method was applicable only to time and prediction. Later on, Hsu and Yang [81] introduced the triangular fuzzy number into the FDM to improve it further, so that the expert opinion is well-presented, and it has been well-practiced in different studies [82]. To avoid the effect of statistical biasness and extreme values, the geometric mean is taken, and the max and min values of the expert opinion are taken as the main points of triangular fuzzy numbers. This method is most suitable for the selection criteria; it is simple and considers the opinion of the experts. The steps of the FDM are as follows:

Step 1: To identify the drivers of KM for healthcare through detailed literature review.

Step 2: After the identification of drivers, “*m*” number of experts, including one medical consultant, two medical officers, and two academicians, got together to evaluate the importance of the driver using the triangular fuzzy numbers. These experts were selected on the basis of their relevant work experience and sound understanding of KM implementation. All of the experts had a minimum of 5 years of work experience and had greater awareness about the significance of KM implementation in the health sector.

Let $w_{j}^{m}=(p_{j}^{m}q_{j}^{m}r_{j}^{m})$ represents the preference of each “mth” decision maker for KM driver j in triangular fuzzy numbers. To accumulate the preferences of all “m” decision makers, Equation (1) will be used:

(1) $$w_{j}=(p_{j}q_{j}r_{j})=\left[p=\min\{p_{ij}\},q_{j}=\frac{1}{m}\sum_{i=1}^{m}q_{ij},r_{j}=\max\{r_{ij}\}\right]$$

where $w_{j}$ shows the accumulation of triangular fuzzy number.

Step 3: The significant drivers are then shortlisted through comparison of their weight with the geometric mean.

(2) $$w_{j}=\frac{p_{j}+q_{j}+r_{j}}{p},j=1,2,3,\ldots ,n$$

where “*w*” shows the cumulative preference of each expert’s KM driver “j”.

If $p_{j}\geq w$ then driver j is selected.

If $p_{j}<w$ then driver j is rejected.

### 3.2. Interpretive Structural Analysis (ISM)

Stage 3: ISM was introduced by Warfield [83], and it is used for developing graphical representations of different variables of a system. It is a technique that analyzes the direct and indirect relationships of variables (drivers), represents them in a hierarchical graphical model [83], and helps is developing a collective understanding of the relationships [84]. The drivers which have a higher impact are at the lower level of the model whereas the ones with lower impact are at the upper level. 

The ISM technique uses the judgment of experts to determine how the variables are related. The structure is then established on the basis of a mutual relationship; the overall structure is derived from the complex set of variables. The relationships and the complexity of the relationship are shown in the form of a digraph [85]. To apply the ISM technique, the following steps need to be performed in proper order:

Step 1: Identifying the drivers of KM for healthcare through literature review.
Step 2: Developing the structural self-interaction matrix (SSIM), which establishes a pair-wise contextual relationship between the drivers.
Step 3: Developing the initial reachability matrix (IRM) by converting the values of SSIM to binary digits (0 and 1).
Step 4: Developing the final reachability matrix (FRM) by removing the transitivity from the IRM.
Step 5: Establishing Level partition to create levels.
Step 6: Convert the level partition into the conical form.
Step 7: Develop the ISM model on the basis of a conical form.
Step 8: Check the model for inconsistency, and to restructure in case of errors.
Step 9: Perform the MICMAC analysis according to the driving and dependence power of each driver.

The details of the steps are given below:

#### 3.2.1. Structural Self-Interaction Matrix (SSIM)

To develop the SSIM, a group of experts was invited through an invitation letter. Initially, twenty invitations were sent to different healthcare professionals with a cover letter describing the basic concept of knowledge management and its necessity to implement in healthcare. Ten experts accepted the invitation of participating in rating drivers supporting KM implementation in healthcare, showing a 50% response rate. Ten experts from public and private hospitals (3 medical consultants, 2 medical officers, 3 head nurses, 2 Pakistan medical and dental council employees) got together and held a brainstorming session. The experts with their opinion and experience helped in developing the contextual relationship between the variables. To develop the contextual relationship, the experts must determine if one variables influences or is influenced by another variable. In SSIM, the relationship between two variables (*i* and *j*) is represented using four symbols, V, A, X, and O.

- V shows that *i* influences *j*
- A shows that *j* influences *i*
- X shows that *i* and *j* both influence each other
- O shows that *i* and *j* are unrelated

The SSIM table of this study is given below in Table 2.

#### 3.2.2. Initial Reachability Matrix (IRM)

After the SSIM, the IRM is developed by taking the values of SSIM as input. The four symbols, V, A, X, and O are converted to 1’s and 0’s by following certain rules. The rules for substituting are as follows:

- If (*i*, *j*) is represented with V in the SSIM, then the entry for (*i*, *j*) in IRM would be 1 and entry for (*j*, *i*) would be 0
- If (*i*, *j*) is represented with A in the SSIM, then the entry for (*i*, *j*) in IRM would be 0 and entry for (*j*, *i*) would be 1
- If (*i*, *j*) is represented with X in the SSIM, then the entry for both (*i*, *j*) and (*j*, *i*) in IRM would be 1
- If (*i*, *j*) is represented with X in the SSIM, then the entry for both (*i*, *j*) and (*j*, *i*) in IRM would be 0

The rules are as shown in Table 3.

The IRM of the study is given in Table 4.

#### 3.2.3. Final Reachability Matrix (FRM)

The FRM is developed after IRM, and the transitivity that exists in IRM are removed. Transitivity refers to the hidden interrelationship that exists between the variables. If there is a relation between X and Y, similarly between Y and Z, then it is considered that there must be a hidden interrelationship between X and Z. The hidden relationship of variables is represented with 1 * in the FRM (Table 5). 

The FMR table gives the driving and the dependence power. The driving power is the capability of a variable affecting other variables. It is calculated by summing up all the ones in the row. The dependence power consists of the variable itself and other variables that may impact it. It is calculated by summing up all the ones in the column. The FRM of this study is given in Table 5.

#### 3.2.4. Level Partitions

The next step is the development of level partition; these levels are developed with the help of three sets (reachability set, antecedent set, and intersection set) derived from the FRM. The reachability set consists of the variables in rows that have 1’s. Similarly, the antecedent set consists of variables in the columns that have 1’s. From these sets the intersection set is derived, which shows the variables that are common to both. The variable for which reachability and intersection are similar forms the first level of the ISM hierarchy model. Once the level is assigned, the number assigned to that variable is removed, and the step is repeated until all variables have been assigned a level. The level partitioning of this study are shown in Table 6, Table 7, Table 8, Table 9, Table 10, Table 11, Table 12, Table 13, Table 14 and Table 15.

#### 3.2.5. ISM Model

**Figure 2:**
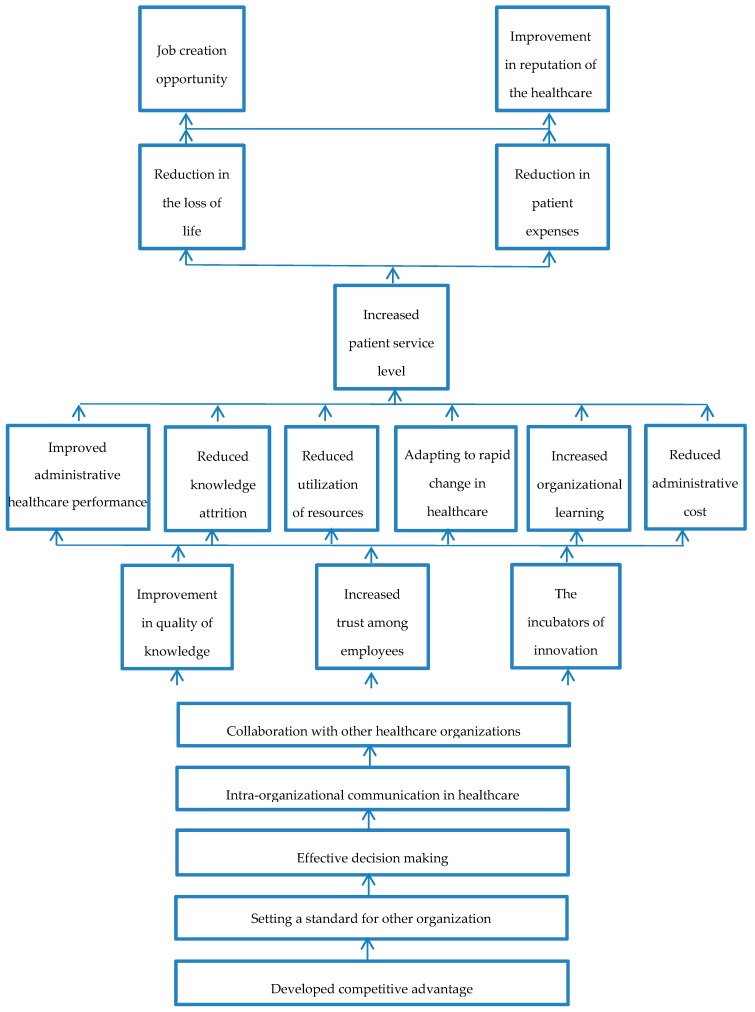
Interpretive Structural Modeling model for drivers.

After level partition the conical matrix is formed, which is developed by correcting the order in the FRM table. The variables of level one are written at the top and the higher levels are written below in proper order. From the conical matrix the diagraph is made, which is the graphical representation of the conical matrix. Diagraph displays nodes with lines showing the relationships and interdependencies. When these nodes are replaced with proper statements they form the ISM model. Figure 2 shows the ISM model of this study given below. 

### 3.3. MICMAC Analysis

MICMAC stands for “Matrices d’Impacts Croises Multiplication Appliqué a un Classement”, which means cross-impact matrix multiplication applied to classification. In the MICMAC analysis, the variables are grouped based on driving and dependence power. These powers are taken from the FRM (Table 5)**.** These variables are then plotted on a graph with four clusters, where the driving power is on Y-axis and dependence power is on the X-axis. The four clusters are called autonomous, dependent, linkage, and independent.

- Autonomous Drivers (Quadrant 1): These are the drivers that have weak driving and dependence power. They generally have little to no influence.
- Dependent Drivers (Quadrant 2): These are the drivers that have weak driving but strong dependence power.
- Linkage Drivers (Quadrant 3): These are the drivers that have strong driving and dependence power. These drivers are very active; an action on one will result in a change in the other.
- Independent Drivers (Quadrant 4): These are the drivers that have strong driving but weak dependence power.

**Figure 3:**
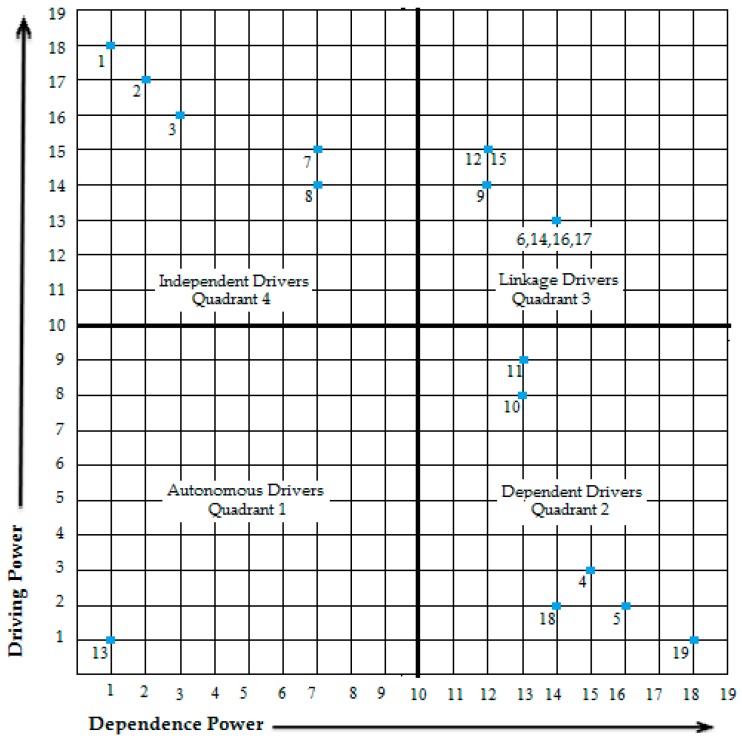
MICMAC analysis showing driving and dependence power.

The drivers of quadrant 3 and 4 are considered to be the main drivers; they have strong driving power. According to the MICMAC analysis of the current study, there is only one autonomous driver in quadrant 1; it is driver 13. The dependent drivers of quadrant 2 are drivers 4, 5, 10, 11, 18, and 19. The linkage drivers of quadrant 3 are drivers 6, 9, 12, 14, 15, 16, and 17. The key drivers are the independent drivers; the drivers in this quadrant (4) are 1, 2, 3, 7, and 8. The MICMAC analysis is given in Figure 3.

## 4. Results and Discussion

The drivers of KM are raising the interests of the healthcare sector and other industries alike. Some of the main drivers are highlighted in this study and analyzed using the ISM technique. The developed ISM model of the current study is showing the interrelationship of the drivers. These drivers are the main reason for the adoption of KM in the healthcare sector. The driving and dependence power give a good idea about the degree of interdependencies among the drivers and will give an insight into the relevant authorities about the benefits of KM in the healthcare of Pakistan. From the ISM and MICMAC techniques, we obtained the following results:

1. Autonomous Drivers: There is only one driver in this quadrant, which is driver 13 (job creation). This driver has weak driving and dependence power, showing that it is relatively less important. The presence of only one enabler in this quadrant proves that the other 18 drivers have more contribution in influencing the system.
2. Dependent Driver: There are six drivers in this quadrant, drivers 4 (increased patient service level), 5 (reduction in the loss of life), 10 (reduced knowledge attrition), 11 (reduced utilization of resources), 18 (reduction in patient expenses), and 19 (improvement in reputation of the healthcare). These drivers have a weak driving but strong dependence power; they cannot influence other drivers.
3. Linkage Drivers: There are seven drivers in this quadrant, drivers 6 (improved administrative healthcare performance), 9 (improvement in quality of knowledge), 12 (increased trust among employees), 14 (adapting to rapid change in healthcare globally), 15 (the incubators of innovation), 16 (increased organizational learning), and 17 (reduced administrative cost). These drivers have strong driving and dependence power. These are amongst the main drivers, the pursuance of one driver will automatically activate the other drivers to help and show benefits.
4. Independent Drivers: These are the key drivers, there are five drivers in this quadrant, drivers 1 (developed competitive advantage), 2 (setting a standard for other organizations), 3 (effective decision making), 7 (intra-organizational communication in healthcare), and 8 (collaboration with other healthcare organizations). These drivers have strong driving but weak dependence power. These are the main reason for Pakistan implementing KM in its health sector.

In the ISM methodology, a group of experts got together and with their judgment determined the interrelationships of the drivers. On the basis of these mutual relationships, an overall structure was formed; the structure is formed by considering the complexity of the relationships between the drivers. The drivers that are at the base of the structure have more impact as compared to those at the top of the structure. According to the current study, the driver that forms the foundation of the structure and is the main reason for implementing KM in the healthcare of Pakistan is developing competitive advantage (driver 1). Due to globalization, the competition has increased significantly in all industries; many organizations are looking to gain a competitive advantage [85,86,87]. The healthcare industry is still far behind; it is also trying to gain a competitive advantage by implementing KM [10,12,35,88]. Developing sustainable competitive advantage as the top-most driver, matched with the earlier studies in KM implementation as du Plessis [57,60], also identified development of sustainable competitive advantage as a major driver in the corporate sector. These results highlight the necessity of a sustainable healthcare system to gain a sustainable competitive advantage. Pakistan over the years had great problems with its healthcare sector, including many diseases and outbreaks [20]. Many policies were developed [22] and increased the budget spending [89] in the sector, but it did not improve the service. Pakistan is now considering the adoption of KM in its healthcare to improve its service and gain sustainable competitive advantage. It will also help Pakistan to compete globally, since international medical tourism is increasing [37], which will be a good opportunity. The implementation of KM will help the healthcare of Pakistan set a standard for healthcare of developing countries and other sector industries. 

The gaining of competitive advantage (driver 1) will lead to setting a standard for other organizations. Competitive advantage involves specialization; it will benefit the healthcare sector and improve patient service. Specialization focuses on best practices, reduction in wasted efforts, better quality of service, and more productivity [90]. It also helps lower the utilization of resources, and due to these benefits competitive advantage helps in setting a standard. The setting of standard for other organizations is like becoming “The big guns for global competition” [91]. By setting a standard, the organizations can define their terms and make the competition follow their methods and practices [91]. If the healthcare of Pakistan implements KM, it will be able to set a standard for itself slowly. The improvement will come over time; eventually, it might become a standard for other developing countries. The healthcare of Pakistan might influence other industries related to healthcare, such as pharmaceuticals, medical equipment manufacturers, medical insurance providers, and others, to adopt KM as well. 

The setting of standards for other organizations (driver 2) further gives rise to effective decision making (driver 3). Setting of standards for other organizations came forward as a major driver in this study, which contradicts the previous studies, however, Yu [64] and Ernst [67] identified KM as a support in effective decision making in their studies, which indicates the importance of KM in supporting decision making in the health sector. A standard can only be set if the decision making is effective. In an organization, the decisions are being made at different levels, e.g., strategic level, managerial level, operational level, and customer level, all done to meet the basic objective. Effective decision making is derived by selecting from alternatives managed well to reach the main objective [92]. Drucker [93] mentioned that effective decision making is a systematic process with clearly defined objectives, and they are required to be adopted sequentially. In the healthcare sector, the decision making at the patient level is crucial, the reason being that the human life is involved. According to a study by Blake [94], it is described that ineffective decision making leads to many errors, deaths, and improper medical prescriptions. The healthcare of Pakistan, with a large infrastructure, is under the control of the government, and is always overcrowded with patients [95,96]. Effective decision making will help in dealing with the patients quickly and effectively, resulting in quick processing of patients and a reduction of overcrowdedness. It will also help in lowering the wastage of time for both patients and doctors alike. 

Effective decision making (driver 3) will result in intra-organizational communication (driver 7). Earlier studies also found intra-organizational communication as an important driver in healthcare [97], and it is very important for effective decision making. In the healthcare sector, a doctor can make a good decision only if he has all the information, which will result in better patient care [8]. In Pakistan, the private sector healthcare has a certain amount of intra-organizational communication. It is, however, not used to share knowledge but just information, and mostly used for administrative purposes, billing, and test results sharing. In the public healthcare sector (Pakistan) there is almost no intra-organizational communication, it is way behind other developed and developing neighboring countries when it comes to technology and communication [98]. All the information shared is paper-based, including the test results, billing, and nursing, they all work independently, unless there is face to face contact. The Government of Pakistan (GoP) has recently started focusing on this issue and has started investing heavily in technology to improve performance [99] and the communication system. The introduction of KM in the healthcare of Pakistan will further increase the intra-organizational communication. 

Intra-organizational communication (driver 7) will encourage collaboration with other organizations (driver 8). If in dealing with a critical patient some information or knowledge is missing, one hospital can contact the other to fill in the gaps of knowledge to ensure better patient dealing. Collaboration with other organizations can remove the rigidity that currently exists in the healthcare of Pakistan; it will help in better service of patients by cross-organizational communication [100]. Dyer [101] says that collaboration between organizations is a potential source of knowledge and it is equally important in all kinds of organizations, especially in knowledge-oriented organizations in the health sector. These days, creating knowledge and its acquisition are more important and can only be done by collaborating with other organizations [102,103,104]. Currently, there is little to no collaboration between the private and public sector healthcare organizations of Pakistan. The introduction of KM will improve this issue over time. 

The collaboration with other organization (driver 7) will give further rise to other drivers, including improvement in the quality of knowledge (driver 9), increased trust among employees (driver 12), and becoming the incubator of innovation (driver 15). If there is collaboration with other organizations then there is an increased level of communication. which will result in the creation of trust between the organization and among the employees [70]. They will interact more and there will be many more opportunities for new researches and innovation. The quality of knowledge will also improve with collaboration. These three drivers will result in other drivers such as improved administrative healthcare performance (driver 6), reduced knowledge attrition (driver 10), reduced utilization of resources (driver 11), adapting to rapid change in healthcare globally (driver 14), increased organizational learning (driver 16), and reduced administrative cost (driver 17).

The above-mentioned drivers will result in the improved patient service level (driver 4). Improved patient service level will reduce the loss of life (driver 5) and patient expenses (driver 18). These drivers, in return, will improve the reputation of the healthcare sector (driver 19) and create new job opportunities (driver 13). The findings of the last level of the ISM model highlighted improved patient service level as the least important driver, however, Winkelman and Choo [105] found that improved patient service level is one of the main motivational drivers for KM implementation in healthcare, especially for the treatment of chronic diseases, which is inconsistent in the current study. 

The difference in results on drivers of KM implementation in healthcare is due to different reasons, e.g., change in context, healthcare policy, rules and regulations, and KM maturity. However, these drivers make KM implementation viable for the sake of healthcare sustainability. The findings of this study negated the earlier findings and one important aspect of the triple-A concept of a sustainable healthcare system “affordability”, as patients are willing to pay more for improved healthcare services, which makes KM implementation inevitable. 

## 5. Conclusions

The healthcare industry is a knowledge-based business in which doctors process about two million pieces of information to manage their patients [53]. At the same time, the generation of knowledge in the healthcare industry is very high; it is estimated to increase four times over the lifetime of a healthcare professional. The professionals have to keep updated because patients, due to the internet, have access to a large amount of information and demand better healthcare service. Patients these days are well educated and aware of the healthcare possibilities and treatment options. To overcome these challenges, doctors need to have access to quality knowledge, which can be provided by KM. The implementation of KM would also provide a healthcare organization with a sustainable competitive advantage. 

There are many drivers of KM in the healthcare industry. It is important for healthcare organizations to understand how to utilize them under which situations. Focusing on the drivers in the order given in the study will assist in understanding the drivers of KM. This study used the ISM and MICMAC approaches to identify the drivers of KM in the healthcare of Pakistan and give its realistic representation. The main contribution of this paper is that it develops the relationships between the drivers of KM in healthcare through a single systemic framework; it will help the decision makers in better understanding the situation. 

This study has identified 19 drivers, and they have been partitioned into ten levels based on their impact. According to the results, the most important drivers to implement KM in the healthcare of Pakistan are gaining a competitive advantage (driver 1), setting a standard for other organizations (driver 2), effective decision making (driver 3), intra-organizational communication (driver 7), and collaboration with other healthcare organizations (driver 8). These are independent drivers; they have strong driving power and weak dependence power. It is due to these drivers that GoP is implementing KM in its healthcare sector. 

There are two main contributions of this study; firstly, theoretically, this study adds to the KM literature in the Pakistani context, especially in healthcare, by identifying the drivers for KM in the healthcare sector of Pakistan. Secondly, by using the ISM and MICMAC approaches, the interrelationships were defined. Therefore, this study can serve as a benchmark by providing a foundation for advancing studies about KM implementation in the healthcare sector. This study shows how a chain of drivers will emerge to show the benefits of implementing KM in the healthcare. It will give the healthcare sector competitive advantage (driver 1), resulting in setting standards for other sectors (driver 2) and effective decision making (driver 3).

The limitation of this study is that only 19 drivers were considered for KM in the healthcare of Pakistan. In the future, more drivers may arise or can be considered for potential studies. The study used expert opinions; there is a possibility they change their opinions in the future. This study is only relevant to the situation of Pakistan and needs validation in other contexts. 

In the future there are opportunities for more researches, as in this research the ISM and MICMAC techniques are used. It gives the relationship model of KM drivers in the healthcare of Pakistan. This model is not statistically validated. It can be validated using the Structural equation modeling (SEM), also known as the linear structural relationship approach. SEM has the ability to test and validate hypothetical models. Since SEM does not have the ability to develop a structure-based model, the ISM technique is used to develop the structure of different variables affecting a system. This makes ISM a supportive analytical tool.

## Figures and Tables

**Table 1:** Drivers derived from literature review.

No	Drivers	References
1	Developed competitive advantage	[57,60,65,66]
2	Setting a standard for other organizations	[61]
3	Effective decision making	[57,62,64,67]
4	Increased patient service level	[62,63,68,69]
5	Reduction in the loss of life	Recommended by Group of Experts
6	Improved administrative healthcare performance	[57,62,63]
7	Intra-organizational communication in healthcare	[57,60,61]
8	Collaboration with other healthcare organizations	[57,58,59,60,70]
9	Improvement in quality of knowledge	[57,61,71]
10	Reduced knowledge attrition	[57,58,72]
11	Reduced utilization of resources	[63,73]
12	Increased trust among employees	[59,61,63]
13	Job creation opportunity	[62,63,73]
14	Adapting to rapid change in healthcare globally	[58,71,74]
15	The incubators of innovation	[58,60,62,75]
16	Increased organizational learning	[59,61,62]
17	Reduced administrative cost	[62,63,64,76]
18	Reduction in patient expenses	[62,64,76]
19	Improvement in the reputation of the healthcare	[63,77]

**Table 2:** Structural Self-Interaction Matrix for drivers.

Drivers		1	2	3	4	5	6	7	8	9	10	11	12	13	14	15	16	17	18	19
1	Developed competitive advantage		V	V	V	V	V	V	O	V	V	V	O	O	V	V	V	V	O	V
2	Setting a standard for other organizations			V	V	V	V	V	V	V	V	O	O	O	V	V	V	O	O	V
3	Effective decision making				V	V	V	V	O	V	V	V	O	O	O	V	V	V	O	V
4	Increased patient service level					V	A	A	O	O	O	O	O	O	A	A	A	O	O	V
5	Reduction in the loss of life						A	O	A	O	A	A	O	O	O	A	O	O	O	V
6	Improved administrative healthcare performance							A	O	V	V	V	A	O	X	V	X	V	O	V
7	Intra-organizational communication in healthcare								V	V	V	O	X	O	O	V	O	O	V	O
8	Collaboration with other healthcare organizations									V	V	V	O	O	V	V	O	O	V	V
9	Improvement in quality of knowledge										V	V	X	O	V	A	V	O	O	O
10	Reduced knowledge attrition											O	O	O	V	A	V	V	O	O
11	Reduced utilization of resources												O	O	V	A	O	V	V	O
12	Increased trust among employees													O	O	X	V	O	O	O
13	Job creation opportunity														O	O	O	O	O	O
14	Adapting to rapid change in healthcare globally															A	V	A	O	V
15	The incubators of innovation																V	O	V	V
16	Increased organizational learning																	V	O	V
17	Reduced administrative cost																		O	O
18	Reduction in patient expenses																			V
19	Improvement in the reputation of the healthcare																			

**Table 3:** Initial Reachability Matrix (IRM) rules.

Structural Self-Interaction Matrix	Initial Reachability Matrix	
Element (*i*, *j*)	Element (*i*, *j*)	Element (*j*, *i*)
V	1	0
A	0	1
X	1	1
O	0	0

**Table 4:** Initial reachability matrix for drivers of knowledge management in the healthcare of Pakistan.

No.	1	2	3	4	5	6	7	8	9	10	11	12	13	14	15	16	17	18	19
1	1	1	1	1	1	1	1	0	1	1	1	0	0	1	1	1	1	0	1
2	0	1	1	1	1	1	1	1	1	1	0	0	0	1	1	1	0	0	1
3	0	0	1	1	1	1	1	0	1	1	1	0	0	0	1	1	1	0	1
4	0	0	0	1	1	0	0	0	0	0	0	0	0	0	0	0	0	0	1
5	0	0	0	0	1	0	0	0	0	0	0	0	0	0	0	0	0	0	1
6	0	0	0	1	1	1	0	0	1	1	1	0	0	1	1	1	1	0	1
7	0	0	0	1	0	1	1	1	1	1	0	1	0	0	1	0	0	1	0
8	0	0	0	0	1	0	0	1	1	1	1	0	0	1	1	0	0	1	1
9	0	0	0	0	0	0	0	0	1	1	1	1	0	1	0	1	0	0	0
10	0	0	0	0	1	0	0	0	0	1	0	0	0	1	0	1	1	0	0
11	0	0	0	0	1	0	0	0	0	0	1	0	0	1	0	0	1	1	0
12	0	0	0	0	0	1	1	0	1	0	0	1	0	0	1	1	0	0	0
13	0	0	0	0	0	0	0	0	0	0	0	0	1	0	0	0	0	0	0
14	0	0	0	1	0	1	0	0	0	0	0	0	0	1	0	1	0	0	1
15	0	0	0	1	1	0	0	0	1	1	1	1	0	1	1	1	0	1	1
16	0	0	0	1	0	1	0	0	0	0	0	0	0	0	0	1	1	0	1
17	0	0	0	0	0	0	0	0	0	0	0	0	0	1	0	0	1	0	0
18	0	0	0	0	0	0	0	0	0	0	0	0	0	0	0	0	0	1	1
19	0	0	0	0	0	0	0	0	0	0	0	0	0	0	0	0	0	0	1

**Table 5:** Final reachability matrix for drivers.

Drivers	1	2	3	4	5	6	7	8	9	10	11	12	13	14	15	16	17	18	19	Driv. Power
**1**	1	1	1	1	1	1	1	1 *	1	1	1	1 *	0	1	1	1	1	1 *	1	18
**2**	0	1	1	1	1	1	1	1	1	1	1 *	1 *	0	1	1	1	1 *	1 *	1	17
**3**	0	0	1	1	1	1	1	1 *	1	1	1	1 *	0	1 *	1	1	1	1 *	1	16
**4**	0	0	0	1	1	0	0	0	0	0	0	0	0	0	0	0	0	0	1	3
**5**	0	0	0	0	1	0	0	0	0	0	0	0	0	0	0	0	0	0	1	2
**6**	0	0	0	1	1	1	0	0	1	1	1	1 *	0	1	1	1	1	1 *	1	13
**7**	0	0	0	1	1 *	1	1	1	1	1	1 *	1	0	1 *	1	1 *	1 *	1	1 *	15
**e**	0	0	0	1 *	1	1 *	0	1	1	1	1	1 *	0	1	1	1 *	1 *	1	1	14
**9**	0	0	0	1 *	1 *	1 *	1 *	0	1	1	1	1	0	1	1 *	1	1 *	1 *	1 *	14
**10**	0	0	0	1 *	1	1 *	0	0	0	1	0	0	0	1	0	1	1	0	1 *	8
**11**	0	0	0	1 *	1	1 *	0	0	0	0	1	0	0	1	0	1 *	1	1	1 *	9
**12**	0	0	0	1 *	1 *	1	1	1 *	1	1 *	1 *	1	0	1 *	1	1	1 *	1 *	1 *	15
**13**	0	0	0	0	0	0	0	0	0	0	0	0	1	0	0	0	0	0	0	1
**14**	0	0	0	1	1 *	1	0	0	1 *	1 *	1 *	1 *	0	1	1 *	1	1 *	1 *	1	13
**15**	0	0	0	1	1	1 *	1 *	1 *	1	1	1	1	0	1	1	1	1 *	1	1	15
**16**	0	0	0	1	1 *	1	0	0	1 *	1 *	1 *	1 *	0	1 *	1 *	1	1	1 *	1	13
**17**	0	0	0	1 *	1 *	1 *	0	0	1 *	1 *	1 *	1 *	0	1	1 *	1 *	1	1 *	1 *	13
**18**	0	0	0	0	0	0	0	0	0	0	0	0	0	0	0	0	0	1	1	2
**19**	0	0	0	0	0	0	0	0	0	0	0	0	0	0	0	0	0	0	1	1
**Dep. power**	1	2	3	15	16	14	7	7	12	13	13	12	1	14	12	14	14	14	18	202

Dep. = Dependence, Driv. = Driving, and after removing the transitivity, the hidden relationship is denoted by 1 *.

**Table 6:** Drivers—level 1.

Drivers	Reachability Sets	Antecedent Set	Intersections	Levels
1	1, 2, 3, 4, 5, 6, 7, 8, 9, 10, 11, 12, 14, 15, 16, 17, 18, 19	1	1	
2	2, 3, 4, 5, 6, 7, 8, 9, 10, 11, 12, 14, 15, 16, 17, 18, 19	1, 2	2	
3	3, 4, 5, 6, 7, 8, 9, 10, 11, 12, 14, 15, 16, 17, 18, 19	1, 2, 3	3	
4	4, 5, 19	1, 2, 3, 4, 6, 7, 8, 9, 10, 11, 12, 14, 15, 16, 17	4	
5	5, 19	1, 2, 3, 4, 5, 6, 7, 8, 9, 10, 11, 12, 14, 15, 16, 17	5	
6	4, 5, 6, 9, 10, 11, 12, 14, 15, 16, 17, 18, 19	1, 2, 3, 6, 7, 8, 9, 10, 11, 12, 14, 15, 16, 17	6, 9, 10, 11, 12, 14, 15, 16, 17	
7	4, 5, 6, 7, 8, 9, 10, 11, 12, 14, 15, 16, 17, 18, 19	1, 2, 3, 7, 9, 12, 15	7, 9, 12, 15	
8	4, 5, 6, 8, 9, 10, 11, 12, 14, 15, 16, 17, 18, 19	1, 2, 3, 7, 8, 12, 15	8, 12, 15	
9	4, 5, 6, 7, 9, 10, 11, 12, 14, 15, 16, 17, 18, 19	1, 2, 3, 6, 7, 8, 9, 12, 14, 15, 16, 17	6, 7, 9, 12, 14, 15, 16, 17	
10	4, 5, 6, 10, 14, 16, 17, 19	1, 2, 3, 6, 7, 8, 9, 10, 12, 14, 15, 16, 17	6, 10, 14, 16, 17	
11	4, 5, 6, 11, 14, 16, 17, 18, 19	1, 2, 3, 6, 7, 8, 9, 11, 12, 14, 15, 16, 17	6, 11, 14, 16, 17	
12	4, 5, 6, 7, 8, 9, 10, 11, 12, 14, 15, 16, 17, 18, 19	1, 2, 3, 6, 7, 8, 9, 12, 14, 15, 16, 17	6, 7, 8, 9, 12, 14, 15, 16, 17	
13	13	13	13	1
14	4, 5, 6, 9, 10, 11, 12, 14, 15, 16, 17, 18, 19	1, 2, 3, 6, 7, 8, 9, 10, 11, 12, 14, 15, 16, 17	6, 9, 10, 11, 12, 14, 15, 16, 17	
15	4, 5, 6, 7, 8, 9, 10, 11, 12, 14, 15, 16, 17, 18, 19	1, 2, 3, 6, 7, 8, 9, 12, 14, 15, 16, 17	6, 7, 8, 9, 12, 14, 15, 16, 17	
16	4, 5, 6, 9, 10, 11, 12, 14, 15, 16, 17, 18, 19	1, 2, 3, 6, 7, 8, 9, 10, 11, 12, 14, 15, 16, 17	6, 9, 10, 11, 12, 14, 15, 16, 17	
17	4, 5, 6, 9, 10, 11, 12, 14, 15, 16, 17, 18, 19	1, 2, 3, 6, 7, 8, 9, 10, 11, 12, 14, 15, 16, 17	6, 9, 10, 11, 12, 14, 15, 16, 17	
18	18, 19	1, 2, 3, 6, 7, 8, 9, 11, 12, 14, 15, 16, 17, 18	18	
19	19	1, 2, 3, 6, 7, 8, 9, 10, 11, 12, 14, 15, 16, 17, 18, 19	19	1

**Table 7:** Drivers—level 2.

Drivers	Reachability Sets	Antecedent Set	Intersections	Levels
1	1, 2, 3, 4, 5, 6, 7, 8, 9, 10, 11, 12, 14, 15, 16, 17, 18	1	1	
2	2, 3, 4, 5, 6, 7, 8, 9, 10, 11, 12, 14, 15, 16, 17, 18	1, 2	2	
3	3, 4, 5, 6, 7, 8, 9, 10, 11, 12, 14, 15, 16, 17, 18	1, 2, 3	3	
4	4, 5	1, 2, 3, 4, 6, 7, 8, 9, 10, 11, 12, 14, 15, 16, 17	4	
5	5	1, 2, 3, 4, 5, 6, 7, 8, 9, 10, 11, 12, 14, 15, 16, 17	5	2
6	4, 5, 6, 9, 10, 11, 12, 14, 15, 16, 17, 18	1, 2, 3, 6, 7, 8, 9, 10, 11, 12, 14, 15, 16, 17	6, 9, 10, 11, 12, 14, 15, 16, 17	
7	4, 5, 6, 7, 8, 9, 10, 11, 12, 14, 15, 16, 17, 18	1, 2, 3, 7, 9, 12, 15	7, 9, 12, 15	
8	4, 5, 6, 8, 9, 10, 11, 12, 14, 15, 16, 17, 18	1, 2, 3, 7, 8, 12, 15	8, 12, 15	
9	4, 5, 6, 7, 9, 10, 11, 12, 14, 15, 16, 17, 18	1, 2, 3, 6, 7, 8, 9, 12, 14, 15, 16, 17	6, 7, 9, 12, 14, 15, 16, 17	
10	4, 5, 6, 10, 14, 16, 17	1, 2, 3, 6, 7, 8, 9, 10, 12, 14, 15, 16, 17	6, 10, 14, 16, 17	
11	4, 5, 6, 11, 14, 16, 17, 18	1, 2, 3, 6, 7, 8, 9, 11, 12, 14, 15, 16, 17	6, 11, 14, 16, 17	
12	4, 5, 6, 7, 8, 9, 10, 11, 12, 14, 15, 16, 17, 18	1, 2, 3, 6, 7, 8, 9, 12, 14, 15, 16, 17	6, 7, 8, 9, 12, 14, 15, 16, 17	
14	4, 5, 6, 9, 10, 11, 12, 14, 15, 16, 17, 18	1, 2, 3, 6, 7, 8, 9, 10, 11, 12, 14, 15, 16, 17	6, 9, 10, 11, 12, 14, 15, 16, 17	
15	4, 5, 6, 7, 8, 9, 10, 11, 12, 14, 15, 16, 17, 18	1, 2, 3, 6, 7, 8, 9, 12, 14, 15, 16, 17	6, 7, 8, 9, 12, 14, 15, 16, 17	
16	4, 5, 6, 9, 10, 11, 12, 14, 15, 16, 17, 18	1, 2, 3, 6, 7, 8, 9, 10, 11, 12, 14, 15, 16, 17	6, 9, 10, 11, 12, 14, 15, 16, 17	
17	4, 5, 6, 9, 10, 11, 12, 14, 15, 16, 17, 18	1, 2, 3, 6, 7, 8, 9, 10, 11, 12, 14, 15, 16, 17	6, 9, 10, 11, 12, 14, 15, 16, 17	
18	18	1, 2, 3, 6, 7, 8, 9, 11, 12, 14, 15, 16, 17, 18	18	2

**Table 8:** Drivers—level 3.

Drivers	Reachability Sets	Antecedent Set	Intersections	Levels
1	1, 2, 3, 4, 6, 7, 8, 9, 10, 11, 12, 14, 15, 16, 17	1	1	
2	2, 3, 4, 6, 7, 8, 9, 10, 11, 12, 14, 15, 16, 17	1, 2	2	
3	3, 4, 6, 7, 8, 9, 10, 11, 12, 14, 15, 16, 17	1, 2, 3	3	
4	4	1, 2, 3, 4, 6, 7, 8, 9, 10, 11, 12, 14, 15, 16, 17	4	3
6	4, 6, 9, 10, 11, 12, 14, 15, 16, 17	1, 2, 3, 6, 7, 8, 9, 10, 11, 12, 14, 15, 16, 17	6, 9, 10, 11, 12, 14, 15, 16, 17	
7	4, 6, 7, 8, 9, 10, 11, 12, 14, 15, 16, 17	1, 2, 3, 7, 9, 12, 15	7, 9, 12, 15	
8	4, 6, 8, 9, 10, 11, 12, 14, 15, 16, 17	1, 2, 3, 7, 8, 12, 15	8, 12, 15	
9	4, 6, 7, 9, 10, 11, 12, 14, 15, 16, 17	1, 2, 3, 6, 7, 8, 9, 12, 14, 15, 16, 17	6, 7, 9, 12, 14, 15, 16, 17	
10	4, 6, 10, 14, 16, 17	1, 2, 3, 6, 7, 8, 9, 10, 12, 14, 15, 16, 17	6, 10, 14, 16, 17	
11	4, 6, 11, 14, 16, 17	1, 2, 3, 6, 7, 8, 9, 11, 12, 14, 15, 16, 17	6, 11, 14, 16, 17	
12	4, 6, 7, 8, 9, 10, 11, 12, 14, 15, 16, 17	1, 2, 3, 6, 7, 8, 9, 12, 14, 15, 16, 17	6, 7, 8, 9, 12, 14, 15, 16, 17	
14	4, 6, 9, 10, 11, 12, 14, 15, 16, 17	1, 2, 3, 6, 7, 8, 9, 10, 11, 12, 14, 15, 16, 17	6, 9, 10, 11, 12, 14, 15, 16, 17	
15	4, 6, 7, 8, 9, 10, 11, 12, 14, 15, 16, 17	1, 2, 3, 6, 7, 8, 9, 12, 14, 15, 16, 17	6, 7, 8, 9, 12, 14, 15, 16, 17	
16	4, 6, 9, 10, 11, 12, 14, 15, 16, 17	1, 2, 3, 6, 7, 8, 9, 10, 11, 12, 14, 15, 16, 17	6, 9, 10, 11, 12, 14, 15, 16, 17	
17	4, 6, 9, 10, 11, 12, 14, 15, 16, 17	1, 2, 3, 6, 7, 8, 9, 10, 11, 12, 14, 15, 16, 17	6, 9, 10, 11, 12, 14, 15, 16, 17	

**Table 9:** Drivers—level 4.

Drivers	Reachability Sets	Antecedent Set	Intersections	Levels
1	1, 2, 3, 6, 7, 8, 9, 10, 11, 12, 14, 15, 16, 17	1	1	
2	2, 3, 6, 7, 8, 9, 10, 11, 12, 14, 15, 16, 17	1, 2	2	
3	3, 6, 7, 8, 9, 10, 11, 12, 14, 15, 16, 17	1, 2, 3	3	
6	6, 9, 10, 11, 12, 14, 15, 16, 17	1, 2, 3, 6, 7, 8, 9, 10, 11, 12, 14, 15, 16, 17	6, 9, 10, 11, 12, 14, 15, 16, 17	4
7	6, 7, 8, 9, 10, 11, 12, 14, 15, 16, 17	1, 2, 3, 7, 9, 12, 15	7, 9, 12, 15	
8	6, 8, 9, 10, 11, 12, 14, 15, 16, 17	1, 2, 3, 7, 8, 12, 15	8, 12, 15	
9	6, 7, 9, 10, 11, 12, 14, 15, 16, 17	1, 2, 3, 6, 7, 8, 9, 12, 14, 15, 16, 17	6, 7, 9, 12, 14, 15, 16, 17	
10	6, 10, 14, 16, 17	1, 2, 3, 6, 7, 8, 9, 10, 12, 14, 15, 16, 17	6, 10, 14, 16, 17	4
11	6, 11, 14, 16, 17	1, 2, 3, 6, 7, 8, 9, 11, 12, 14, 15, 16, 17	6, 11, 14, 16, 17	4
12	6, 7, 8, 9, 10, 11, 12, 14, 15, 16, 17	1, 2, 3, 6, 7, 8, 9, 12, 14, 15, 16, 17	6, 7, 8, 9, 12, 14, 15, 16, 17	
14	6, 9, 10, 11, 12, 14, 15, 16, 17	1, 2, 3, 6, 7, 8, 9, 10, 11, 12, 14, 15, 16, 17	6, 9, 10, 11, 12, 14, 15, 16, 17	4
15	6, 7, 8, 9, 10, 11, 12, 14, 15, 16, 17	1, 2, 3, 6, 7, 8, 9, 12, 14, 15, 16, 17	6, 7, 8, 9, 12, 14, 15, 16, 17	
16	6, 9, 10, 11, 12, 14, 15, 16, 17	1, 2, 3, 6, 7, 8, 9, 10, 11, 12, 14, 15, 16, 17	6, 9, 10, 11, 12, 14, 15, 16, 17	4
17	6, 9, 10, 11, 12, 14, 15, 16, 17	1, 2, 3, 6, 7, 8, 9, 10, 11, 12, 14, 15, 16, 17	6, 9, 10, 11, 12, 14, 15, 16, 17	4

**Table 10:** Drivers—level 5.

Drivers	Reachability Sets	Antecedent Set	Intersections	Levels
1	1, 2, 3, 7, 8, 9, 12, 15	1	1	
2	2, 3, 7, 8, 9, 12, 15	1, 2	2	
3	3, 7, 8, 9, 12, 15	1, 2, 3	3	
7	7, 8, 9, 12, 15	1, 2, 3, 7, 9, 12, 15	7, 9, 12, 15	
8	8, 9, 12, 15	1, 2, 3, 7, 8, 12, 15	8, 12, 15	
9	7, 9, 12, 15	1, 2, 3, 7, 8, 9, 12, 15	7, 9, 12, 15	5
12	7, 8, 9, 12, 15	1, 2, 3, 7, 8, 9, 12, 15	7, 8, 9, 12, 15	5
15	7, 8, 9, 12, 15	1, 2, 3, 7, 8, 9, 12, 15	7, 8, 9, 12, 15	5

**Table 11:** Drivers—level 6.

Drivers	Reachability Sets	Antecedent Set	Intersections	Levels
1	1, 2, 3, 7, 8	1	1	
2	2, 3, 7, 8	1, 2	2	
3	3, 7, 8	1, 2, 3	3	
7	7, 8	1, 2, 3, 7	7	
8	8	1, 2, 3, 7, 8,	8	6

**Table 12:** Drivers—level 7.

Drivers	Reachability Sets	Antecedent Set	Intersections	Levels
1	1, 2, 3, 7	1	1	
2	2, 3, 7	1, 2	2	
3	3, 7	1, 2, 3	3	
7	7	1, 2, 3, 7	7	7

**Table 13:** Drivers—level 8.

Drivers	Reachability Sets	Antecedent Set	Intersections	Levels
1	1, 2, 3	1	1	
2	2, 3	1, 2	2	
3	3	1, 2, 3	3	8

**Table 14:** Drivers—level 9.

Drivers	Reachability Sets	Antecedent Set	Intersections	Levels
1	1, 2	1	1	
2	2	1, 2	2	9

**Table 15:** Drivers—level 10.

Drivers	Reachability Sets	Antecedent Set	Intersections	Levels
1	1	1	1	10
